# Targeting COPD with PLGA-Based Nanoparticles: Current Status and Prospects

**DOI:** 10.1155/2022/5058121

**Published:** 2022-03-11

**Authors:** Juhi Saxena, Monish Bisen, Aditya Misra, Vijay Kumar Srivastava, Sanket Kaushik, Arif Jamal Siddiqui, Neetu Mishra, Abhijeet Singh, Anupam Jyoti

**Affiliations:** ^1^Department of Biotechnology, University Institute of Biotechnology, Chandigarh University, Mohali, Punjab 140413, India; ^2^Faculty of Applied Sciences and Biotechnology, Shoolini University of Biotechnology and Management Sciences, Bajhol, Solan, Himachal Pradesh 173229, India; ^3^Amity Institute of Biotechnology, Amity University Rajasthan, Amity Education Valley, Kant Kalwar, NH-11C, Jaipur-Delhi Highway, Jaipur, India; ^4^Department of Biology, College of Science, University of Ha'il, Ha'il, P.O. Box 2440, Saudi Arabia; ^5^Symbiosis School of Biological Sciences, Symbiosis International (Deemed University), Pune, Maharashtra 412115, India; ^6^Department of Biosciences, Manipal University Jaipur, Rajasthan 303007, India

## Abstract

Chronic obstructive pulmonary disease (COPD) is pulmonary emphysema characterized by blockage in the airflow resulting in the long-term breathing problem, hence a major cause of mortality worldwide. Excessive generation of free radicals and the development of chronic inflammation are the major two episodes underlying the pathogenesis of COPD. Currently used drugs targeting these episodes including anti-inflammatory, antioxidants, and corticosteroids are unsafe, require high doses, and pose serious side effects. Nanomaterial-conjugated drugs have shown promising therapeutic potential against different respiratory diseases as they are required in small quantities which lower overall treatment costs and can be effectively targeted to diseased tissue microenvironment hence having minimal side effects. Poly lactic-co-glycolic acid (PLGA) nanoparticles (NPs) are safe as their breakdown products are easily metabolized in the body. Drugs loaded on the PLGA NPs have been shown to be promising agents as anticancer, antimicrobial, antioxidants, and anti-inflammatory. Surface modification of PLGA NPs can further improve their mechanical properties, drug loading potential, and pharmacological activities. In the present review, we have presented a brief insight into the pathophysiological mechanism underlying COPD and highlighted the role, potential, and current status of PLGA NPs loaded with drugs in the therapy of COPD.

## 1. Introduction

Chronic obstructive pulmonary disease (COPD) is a major public health problem contributing to the third most common cause of death globally [1]. Incidences of COPD are prevalent in low-income countries or poorly developed nations leading to ill health and compromised quality of life. The major risk factors include tobacco smoke, biomass, pollution, and a history of tuberculosis infection. The underlying cause of COPD-mediated airway damage includes excessive generation of free radicals and chronic inflammation in the lungs leading to the obstruction in airways and hence experience of difficulty in breathing [2]. The problem is further complicated in patients having pathology of COPD along with cardiovascular complications, resulting in poor patient outcomes [3]. Currently available therapeutic approaches include the use of bronchodilators, cessation of smoking, administration of antibiotics, and corticosteroids. Long-acting *β*2-adrenergic receptor agonists and long-acting muscarinic acetylcholine receptor antagonists are used in combination as an inhaler to relieve the symptoms [4]. As these drugs do not target inflammation, their use is limited to the clinical management of COPD. Corticosteroids showing the promising result as anti-inflammatory fail to be effective as inflammation episode in COPD is exacerbated [5]. Anti-inflammatory drugs including roflumilast, a phosphodiesterase-4 inhibitor, have shown promising result in improving lung function and preventing exacerbations in COPD. However, serious adverse effects have restricted its applications in the treatment of COPD [6]. Hence, there is the greatest unmet therapeutic need to develop a safe and effective drug for the clinical management of COPD.

Smart drug delivery systems (DDS) are emerging and alternative therapeutic strategies. Polymeric nanoparticles (NPs) are promising tools for DDS against multiple diseases as they strongly improve the potency of many existing drugs [7]. PLGA, a synthetic polymer, is eclectic for nanodrug formulations for the treatment of chronic respiratory diseases generally recognized as safe by the Food and Drug Administration (FDA). PLGA is the best choice in the design of nanodrug delivery systems for the treatment of various diseases. They have applications in cancer therapy [8], wound healing [9], antimicrobial [10], antioxidant [11], and anti-inflammatory activities [12]. The key products released after PLGA breakdown are water and carbon dioxide, which gets easily removed through the citric acid cycle in the body [13]. PLGA exhibits a remarkable set of favorable features like high biocompatibility, biodegradable nature, adaptable degradation, and tunable mechanical properties. Its hydrophobic nature can be altered through surface modification strategies for improved biological activities. PLGA NPs loaded with the drug have been used in mouse models where they maintained dug concentration for a longer duration in the blood and shows good stability [14]. Drug-loaded PLGA NPs have a slightly larger size range. Therefore, a smaller size of PLGA-based NPs during synthesis is recommended as it improves the cellular uptake of the drug. The main challenge for the exploitation of PLGA NPs as drug carriers is its dissociation and hydrophobic nature. To enhance polymer-drug stability and its bioefficacy, modifications in glycolic and lactic acid ratio and surface properties are to be carried out followed by drug conjugation [15]. Small RNA molecules and drug conjugates have been developed to treat chronic respiratory diseases which can be loaded onto PLGA NP for improved pharmacological actions [16]. PLGA NPs bear the potential for effective and safe therapy of several pulmonary diseases.

Here, we present a comprehensive overview of PLGA NPs and their properties, routes of synthesis, controlling parameters, potential as drug carriers, role, and prospects in the pathophysiology and progression of COPD.

## 2. COPD

COPD is the gradual decrease in lung function marked by an irreversible obstruction in the airways primarily due to the chronic inflammatory insult of the peripheral airways [17]. This disease is characterized by emphysema which includes damage to the alveoli and chronic bronchitis having obstructive airflow, resulting in short breaths during vigorous physical activity. COPD is, therefore, a serious public health concern and assessed to be the third major cause of death globally [2].

The disease involves continuous aging and reduction in the supporting tissues of the lungs which turns to peak in old people 65 years of age. Active smoking or passive exposure to smoke stands out to be the prominent cause of COPD as it deteriorates the state with oxidative stress and inflammation leading to airway damage in the lungs. In the majority of cases, cigarette smoking is associated with COPD cases in developed countries, whereas in poorly developed countries, indoor pollutants are the major reason for COPD [18]. The main symptoms include difficulty breathing, reduced exercise capacity, weakness, and limited capability to perform routine tasks hampering the quality of life. The additional risk factors could be genetics, epigenetics, history of having tuberculosis, and inhaled pollutants like smoke from burning of fuels and biomass [19]. Gender is no bias in developing COPD, although males are at a higher risk because males are more likely to smoke in comparison to females [20].

### 2.1. Pathophysiology of COPD

**Figure 1:**
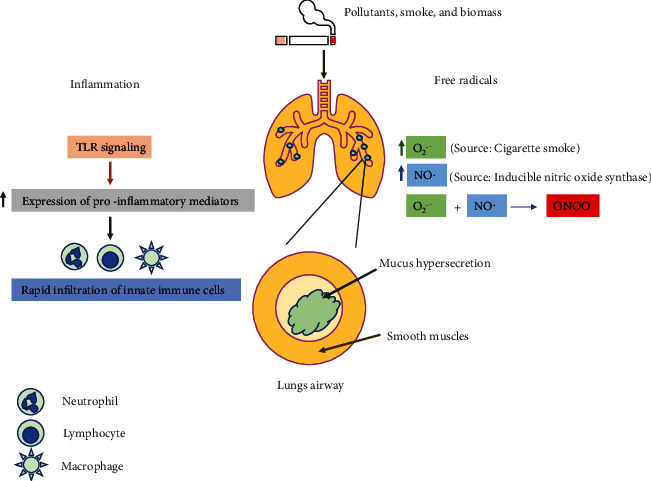
The pathophysiologic processes involved in COPD. Pollutants, cigarette smoke, and biomass induce excessive generation of oxidative and nitrosative stresses including superoxide, nitric oxide, and peroxynitrite radicals. These radicals further activate the p38MAPK and NF-*κ*B proteins which then upregulate the expression of genes and proteins involved in the inflammatory pathway. Additionally, smoke and pollutants also induce TLR signaling and initiate the inflammatory response by augmenting the expression of proinflammatory mediators. This led to the rapid infiltration of innate immune cells including neutrophils, macrophages, and lymphocytes. These cells release many proteases which stimulate mucus hypersecretion in COPD patients.

The underlying process involved in the pathogenesis of COPD includes chronic inflammation and excessive generation of free radicals including oxidative and nitrosative stress (Figure 1).

#### 2.1.1. Chronic Inflammation

Chronic inflammation is the predominant characteristic in COPD affecting the peripheral airway system and pulmonary parenchyma [21]. Upon exposure and inhalation of pollutants, smoke, and biomass, Toll-like receptor-mediated signaling is activated and initiates the inflammatory response by augmenting the expression of proinflammatory mediators. The expression of these mediators is under the tight control of p38 map kinase (MAPK) and transcription factor NF-*κ*B [22, 23]. This led to rapid infiltration of innate immune cells including neutrophils, macrophages, and lymphocytes [24]. In a later time, point, there is an increase in the number and migration of adaptive immune cells including CD4^+^ T helper and CD8^+^ cytotoxic T cells towards the lungs that occurs which further augment the cellular inflammation [25, 26]. In some cases, the patient exhibits elevated numbers of eosinophils in the air passages, a feature related to asthma [27]. Additionally, there is an upregulation and secretion of many proteases from epithelial cells, macrophages, and neutrophils in lung airways which degrade elastin fibers. Also, neutrophil elastases stimulate mucus hypersecretion in COPD patients. Metalloproteinases like MMp9 and MMp12 further lead to elastolysis in lung parenchymatous tissues as observed in emphysema.

Healthy individuals with normal lung functions have increased inflammation in the airway which revealed that this is the normal response by lung mucosa when exposed to inhaled smoke irritants. However, typically in COPD patients, there is an upregulation of multiple inflammatory genes; also, the inflammation gets amplified during mild exacerbations because of a decline in the expression of nuclear enzyme histone deacetylase 2 (HD2) encoded by HDAC2 in epithelial cells and macrophages present in the lungs of patients [28]. Inflammation is further aggravated in COPD patients and is linked with the colonization of *Streptococcus pneumoniae* and *Haemophilus influenzae* in the lower respiratory tract [29, 30]. This led to the defective phagocytosis of bacteria and apoptotic cells by macrophages which results in impaired resolution of lung inflammation [30, 31]. This is one of the reasons that, even after cessation of smoking, the inflammation persists.

#### 2.1.2. Free Radicals

The driving force in the pathophysiology of COPD is elevated oxidative stress and therefore results in varied features in the development of disease [32]. Patients suffering from COPD due to exposure to cigarette smoke develop heavy oxidative stress; this will further increase by the activation of macrophages and neutrophils. Heavy oxidative burden and hyperinflammation result from reactive oxygen species (ROS) which creates complexity in the pathophysiology of COPD. ROS, for instance, activate p38MAPK and NF-*κ*B which then upregulate the expression of genes and proteins involved in the inflammatory pathway. Further, ROS also hinder antiprotease *α*1-antitrypsin which is expressed endogenously, resulting in severe elastolysis. DNA damage due to oxidative stress will be poorly repaired, which otherwise in normal conditions get corrected by DNA repair machinery. The double-stranded DNA breaks fail to get repaired and are likely to increase the chances of lung cancer [33]. COPD patients have autoantibodies due to carbonylation of proteins upon ROS induction; these antibodies circulate in the blood and penetrate the lung mucosa, worsening the lung inflammation (Kirkham 2011). ROS-mediated activation transforming growth factor-*β* (TGF*β*) develops fibrosis in lung tissues. Furthermore, oxidative stress causes a decrease in levels of HD2 which further reduces the responsiveness to corticosteroids [17]. Defective internal antioxidant defense and ROS burden reduce the SIRT1 activity in the patient's lung suffering from COPD and thus hamper autophagy and genomic stability as well as damage the shield against ageing and cellular senescence [34]. Nuclear transcription factor NRF2/NFE2L2 is a key role player in regulating cytoprotective genes and antioxidant balance in response to oxidative stress, but the COPD patients have diminished NRF2 functions. Low activation levels of NRF2 under oxidative stress are due to increased acetylation and reduction in HD2 activity [35].

Currently, there is emerging evidence that there is mitochondrial dysfunction in COPD and mitochondrial ROS leads to a reduction in endogenous ATP and decreased oxidative phosphorylation. By mimicking cigarette smoke in vitro, it has been investigated that there is cellular senescence and ROS overproduction leading to mitochondria fragmentation in COPD patient's epithelial cells [36]. Cigarette smoke is the inducer of mitochondrial autophagy/mitophagy in epithelial cells present in lung tissues. Mitophagy regulator phosphatases and PINK1 protein ultimately led to necroptosis and mitochondrial deficiency [37]. PINk1 knockout mouse models developed by researchers further confirmed that increased levels of PINk1 in COPD result in emphysema and mucous secretion upon long-time cigarette smoke exposure.

### 2.2. Models to Study COPD

The implications of COPD are compounded by the absence of novel drug targets in the area of pulmonary medicine, largely resulting from a lack of knowledge surrounding the progression of the disease. In the later stages of COPD, disease modeling has been difficult as no single in vivo model for COPD has been able to capture the full breadth of the symptoms experienced by the patients [38].

In vitro models facilitate the study of selected physiological processes within a controlled environment before confirming the hypothesis in a more complex in vivo environment. There have been various animal models also which have been tested for the treatment options for COPD, e.g., rat, mouse, guinea pig, hamster, rabbit, and dog models [38]. Many COPD mechanisms have been tested in immortalized cell lines, such as BEAS2B and A549, derived from the human bronchial epithelium and human lung alveolar epithelium, respectively. A549 cell lines are used in many experiments to test the levels of ROS post-COPD. Model cells offer less variability and are easier to maintain and manipulate in culture, while still maintaining lung-derived primary cell features. While choosing a specific model for COPD, it is a necessity to check its pathophysiological and anatomical features [39].

There are various questions that need to be answered while choosing an appropriate model. Does it provide the salient features of the disease, is there a quantifiable and an agreeable change in the state of the disease following application of treatment, to what degree are the changes recapitulated in humans, etc. The value of a model is greatly diminished if it does not show a measurable change following treatment. The translation of these findings in preclinical models to clinical trials is paramount to the process of novel treatment discovery.

## 3. Nanoparticles: Smart Applications in Drug Delivery System

The drug delivery system (DDS) consists either of a formulation or device that releases active molecules into the human systems. This will not only improve bioefficacy but also add to safety parameters by controlling the amount of available drug release at the site to penetrate biomembranes and the real time to act upon the therapeutic targets. This system includes the drug molecule along with the carriers to mediate the diffusion and delivery of active ingredients in the human body. DDS allows multiple opportunities for personalization, optimization which rely upon the patient and a particular set of disease. The administration routes and active substances to be delivered are therefore significant factors when targeting and treating disease. The invasive routes are associated with a high risk of secondary infections; oral routes have limitations with pH and solubility factors. In the present scenario, the line of chemotherapeutic agents possesses critical toxicity, less specificity, and the emergence of drug resistance with many drug systems. Therefore, to reduce the existing risks and disadvantages associated with the conventional system, the DDS system is now evolving with better adapted alternatives [7, 40, 41]. In this regard, nanomedicine recently has emerged as a path-breaking exploratory area for developing innovative and effective treatment strategies in fatal human diseases. In the last few years, several research investigations based upon NPs as effective agents have gained much attention especially in the area of drug delivery and therapeutics.

With the advancements in technologies and availability of novel tools, the employment of silver and gold nanoparticles, nanotubes, micelles, liposomes, and polymeric nanoparticles as carriers for administering bioactive compounds has been rapidly increased [42, 43]. NPs are prevalent choice-based systems for drug delivery as they not only protect and stabilize the drug but also enhance its targeting to specific tissues. These features improve drug efficacy and reduce underlying side effects. Many studies have focused on nanoencapsulated drugs using NPs which passively increase tissue targeting due to the “greater permeability and retention” [44]. Moreover, these days, NPs have been used to deliver antibiotics, immunomodulatory molecules, DNA vectors, and vaccine components [42]. The physicochemical properties of NPs are detrimental to their interaction with biological systems. Various parameters like chemical composition, shape, size, oxidative potential, zeta potential, and protein surface corona surface influence their intrinsic role along with the extrinsic environment [45].

The influence of drug-loaded NPs on the cellular response can be finely tuned by altering their structure, size, and loading abilities. These features therefore are prominently involved in determining immune activation or deactivation during disease-specific targeting [46]. The vital and distinguishing characteristic of NPs that makes them particularly suited for lung disorders is the nature of nanomaterial used for synthesis. Sometimes, the type of nanomaterial can elicit the cellular toxicity and activation of inflammatory cells. Positively charged NPs induce more immunogenic reactions than negatively charged or neutral NPs [47]. Importantly, the size of NPs influences their cellular uptake, biodistribution, and T cell responses. Small NPs due to their size can escape from antigen-presenting cells at the site of administration and are therefore better suited as carriers or vectors. Moreover, bigger NPs tend to induce more complications by aggravating the cell-mediated immune response specifically to Th1 immune mediator cells [48]. Recently, polymeric NPs have gained focused attention due to their biocompatibility and nonimmunogenicity. Polymeric NPs made up of PLGA improve the bioavailability and potency of many drugs [41].

### 3.1. Polymeric NPs: An Overview

Nanoparticulate delivery structures which are evolved from nanotechnology-based platforms transport drug molecules by nanocarriers having submicron size typically <500 nm. These nanoprototypes have a high volume-to-surface ratio with minimum side effects and can be effectively targeted to diseased tissue microenvironment. Nanocarrier precursor material should meet the criterion for nonimmunogenicity, biodegradability, and biocompatibility [49, 50]. Currently, smart nanostructures can be categorized into inorganic and organic nanocarriers. Their physicochemical characteristics depend mainly on the chemical compositions including inorganic, organic, or hybrid; dimensions whether small or large in sizes; shapes or arrangements like star, rod, sphere, branched or hyperbranched, and lamellar or multilamellar structures; surface charges, functional groups, and coating materials. Based on the literature survey, in comparison to inorganic and metallic nanoparticles, some drug formulations with organic polymer-based nanoparticles received FDA and European Medicines Agency (EMA) approval and have proven to be more successful as effective drug carriers in humans and exhibited excellent safety profiles during clinical investigations [42].

**Figure 2:**
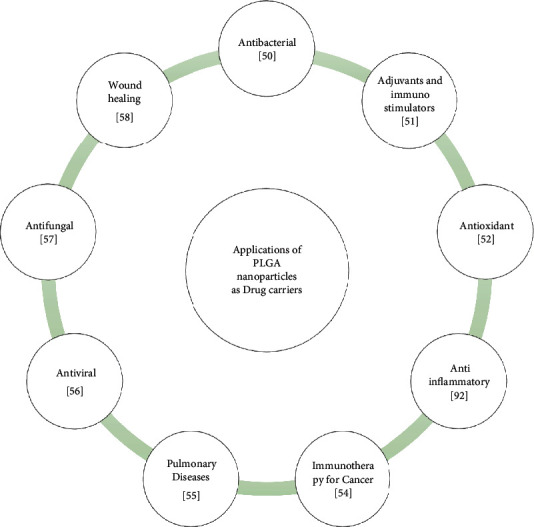
Applications of drug-loaded PLGA NP. PLGA NP loaded with drugs have been shown to be promising agents as antibacterial, adjuvant and immune-stimulator, antioxidant, anti-inflammatory, anticancer, antiviral, antifungal, and wound healer.

Macromolecules formed through covalent linkages between monomers constitute a polymer. Based upon the arrangements of monomeric units, polymers can be divided into the branched or linear chains. Functional groups present in each monomer will direct the linkages and thereby control specific properties. Polymers are marked by versatility and can be easily customized as per the requirements of the researcher. The polymeric nanoparticle which ranges in size from 1 to 1000 nm includes nanospheres or nanocapsules. These polymeric nanoparticles thus differ in their morphological structures. Nanocapsules represent a reservoir system in which drug can be encapsulated in the interior whereas nanospheres represent a matrix system in which drug can be retained either inside or adsorbed on the surface. Among all the biodegradable polymeric materials, the most commonly used for drug delivery systems are PLGA and poly (lactic acid) (PLA) copolymers. PLGA NP finds diverse applications as drug carriers to treat various diseases (Figure 2). In the subsequent sections, we will discuss about the PLGA NPs and their therapeutic role in COPD [42, 45, 58, 59].

### 3.2. PLGA NPs: Properties and Routes for the Synthesis

The biodegradable copolymer PLGA is made up of lactic acid and glycolic acid bonded together with ester linkages. It easily breaks down to water and carbon dioxide which are nontoxic products. Fabrication of PLGA NPs involves two different methods: nanoprecipitation method and solvent-evaporation method (also called as single or double emulsion method).

#### 3.2.1. Size

Currently, nanoparticles employed in therapeutics should lie in the size range of 10–100 nm. PLGA NPs having a size of less than 100 nm can cross mucosal lining and the immune defense barrier. During the passage across membranes, these particles get exposed to multiple barriers before the uptake of lung cells which results in their premature collapse. Sufficiently smaller particles can avoid their deposition in the airways and can traverse through the layer due to minimal steric interference. Rod-shaped and spherical nanoparticles have better penetration as compared to disk- or barrel-shaped nanoparticles [60].

#### 3.2.2. Surface Properties

Nanoparticles exhibit high surface-to-volume ratio as compared to their bulk materials, and thus, it controls their interaction in the biological system. PLGA NPs which get stabilized with polyethylene glycol have an either positive or negative surface which restricts their self to self as well as self to non-self-particle interactions. The larger positive or negative surface charge would increase clearance by macrophages through the reticuloendothelial system. The surface charge not only controls interactions but would also influence distribution along with size-limited locations within the system. So, to have target locations for polymeric nanoparticles, it is imperative to study surface properties with size specificities [61].

#### 3.2.3. Surface Ligands

Targeting ligands facilitates to provide better nanoparticle-cell surface interactions and leads to ultimate cellular location when delivered. For example, specific to lung cells, cell surface receptors, proteins, and small moieties can be ligated which would ease the entry of polymeric nanoparticles at the site of action by receptor-mediated endocytosis. Thus, modifying nanoparticle surface properties with suitable ligands would enhance their cellular uptake [40].

#### 3.2.4. Solvent Evaporation Method for PLGA NP Synthesis

During the solvent evaporation route for PLGA NPs, presynthesized polymer and oil-in-water (o/w) emulsion are required. Initially, the polymer is dissolved in a polar organic solvent followed by dissolution of drug molecules. The most commonly used organic solvents are chloroform, dichloromethane, and ethyl acetate. Recently, ethyl acetate is proven to be better adapted for biomedical applications having less toxicity. The aqueous phase contains surfactants like polyvinyl acetate (PVA). Now, the organic phase is emulsified in an aqueous phase containing surfactant slowly. The emulsion is finally prepared with either homogenization or ultrasonication which yields dispersion of nanodroplets. The polymer solvent is then evaporated at room temperature or by magnetic stirring. After evaporation of the solvent, solid nanoparticles can be washed and collected through centrifugation. Lyophilization or freeze drying can be used for long-term storage of these nanospheres [62].

#### 3.2.5. Nanoprecipitation

The nanoprecipitation method is also known as the solvent displacement method and requires two solvents that are miscible. In the internal phase, the polymer is dissolved in one of the miscible organic solvents like acetonitrile or acetone, and the same solution is added stepwise to an aqueous phase under constant stirring either with a syringe or dropper. Due to the quick addition and diffusion of polymer into an aqueous solution, nanoparticles formed instantly in the form of nanospheres or nanocapsules. Surfactants can also be used during this process for the stability of the suspension. This procedure has an advantage over the emulsion solvent evaporation method as the nanoparticles are of narrow size distribution and have well-defined size ranging from 170 to 200 nm.

These conventional routes for preparation except the emulsion method have few disadvantages as they result in polydispersed spherical particles ranging in size from 150 to 300 nm. Agitation and centrifugation speed also affects the polydispersity. Also, such methods reduce the encapsulation and drug loading efficiency of the particles. Surface coatings of PLGA NPs with polyethylene glycol (PEG) can improve loading and release rate, hence adding to the dynamic applications. PEG is the preferred hydrophilic polymer for surface tuning in PLGA- and PLA-based nanomaterials. This makes up amphiphilic copolymer, wherein hydrophilic PEG shell protects the hydrophobic core containing drug molecules from phagocytes as well as inhibits unwanted protein agglomeration while circulating in the blood [42]. Recently, using microreactors, the physiochemical properties like size, shape, and loading efficiency of PLGA NPs can be precisely fine-tuned which further reduces the batch-to-batch variability. Microreactors in microfluidics utilize either droplet-based systems or continuous laminar flow systems for the synthesis of polymeric nanoparticles [63].

The fundamental reason for using PLGA NPs as DDS is their loading efficiency with varied molecules. The drug release kinetics from encapsulated PLGA NPs depends on polymer molecular weight, storage conditions, surface structure and coating, and lactide to glycolide ratio. The solubility of PLGA NPs is relying mainly upon its monomeric components. If more proportion of glycolic acid is present, the nanoparticles will be soluble in fluorinated solvents like hexafluoroisopropanol. However, if there is a high ratio of lactic acid, then it will lead to more hydrophobic PLGA, which makes PLGA NPs soluble in organic solvents like dichloromethane, chloroform, or acetone. Physiochemical characterization of PLGA NPs can be performed with several advanced techniques. Size can be evaluated by dynamic laser scattering (DLS) and morphology by scanning electron microscopy (SEM), transmission electronic microscopy (TEM), or atomic force microscopy (AFM) [64].

## 4. Pharmacological Actions of PLGA NP-Drug Conjugates for COPD

**Figure 3:**
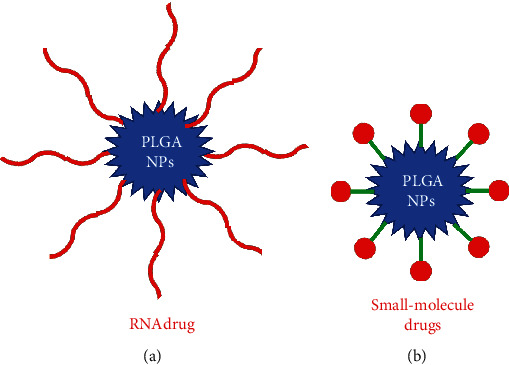
Types of PLGA NP-drug conjugates used in the treatment of COPD. PLGA NPs engineered with RNA and small-molecule drugs have been shown to have a protective role in COPD.

Polymers conjugated with drugs improve their pharmacological activity by enhancing their solubilization, controlled delivery, sustained circulation, reduced immunogenicity, and reduced toxicity [65]. In the following section, we will discuss the PLGA NP–RNA and PLGA NP–small-molecule conjugates with special reference to COPD (Figure 3).

### 4.1. PLGA NP–RNA Conjugates

With the advent of RNA interference (RNAi) technology in 1998, a significant upsurge in the development of RNA-based therapeutics has been driven [66]. A major class of RNA-based therapeutics includes microRNAs (miRNAs) which are widely used to treat respiratory diseases because of their endogenous nature to inhibit the gene expression as well as their tractable approach (Figure 3(a)) [67–69]. miR-146a, a class of miRNA, has a pivotal role in the pathogenesis of COPD [70]. It downregulates interleukin-1 receptor-associated kinase (IRAK-1) expression and thereby inhibits IL-1R signaling, hence having therapeutic value [71]. However, the major bottleneck in using miRNA as a therapeutic agent is its unstable nature and being unable to cross the anionic cell membrane [72]. PLGA NPs coated with cationic materials can adsorb negatively charged RNA molecules. In a study conducted by Mohamed et al. (2019), they have loaded miR-146a onto NPs prepared using PGA-co-PDL to attenuate IRAK-1 and regulate the inflammatory process involved in COPD [73].

Little progress has been achieved in formulating PLGA-miRNAs for clinical applications in COPD. The underlying reason could be the instability of miRNA which led to rapid degradation upon application in humans [74]. Another reason is the immunogenicity of polymer. The presence of antipolymer antibodies has been correlated with the loss of efficacy of polymer-coated therapeutic molecules [75]. Modifications in the encapsulation of miRNAs with PLGA NP are required for enhanced targeting efficacy and reduced off-target effects.

### 4.2. Polymer–Small-Molecule Drug Conjugates

Several pharmacologically active small molecules have been discovered to treat COPD, but their lower permeability across mucus lining limits their use in the clinics [76, 77]. To mitigate this, Vij et al. (2016) have conjugated ibuprofen, an anti-inflammatory drug, with the PLGA-PEG NP to target neutrophil-mediated inflammatory response in COPD [78]. Upon conjugation, ibuprofen attenuated lipopolysaccharide- (LPS-) and cigarette smoke- (CS-) induced lung injury, hence holding promise in clinics. Furthermore, 1,3-di[5-(N-methylene-pyridinium-4-yl)-10,15,20-triphenylporphynato manganese]-benzene tetrachloride (MnPD), an antioxidant, conjugated with PLA NP results in inhibition in the production of ROS as well as IL-8 in COPD [79].

The major challenge of using PLGA-loaded drug molecules is its rapid dissociation and release which reduces the efficiency of polymer carriers. To overcome this, there is an urgent need for modification in cross-linking to enhance the polymer-drug stability and controlled release of the drug under complex biological systems.

**Figure 4:**
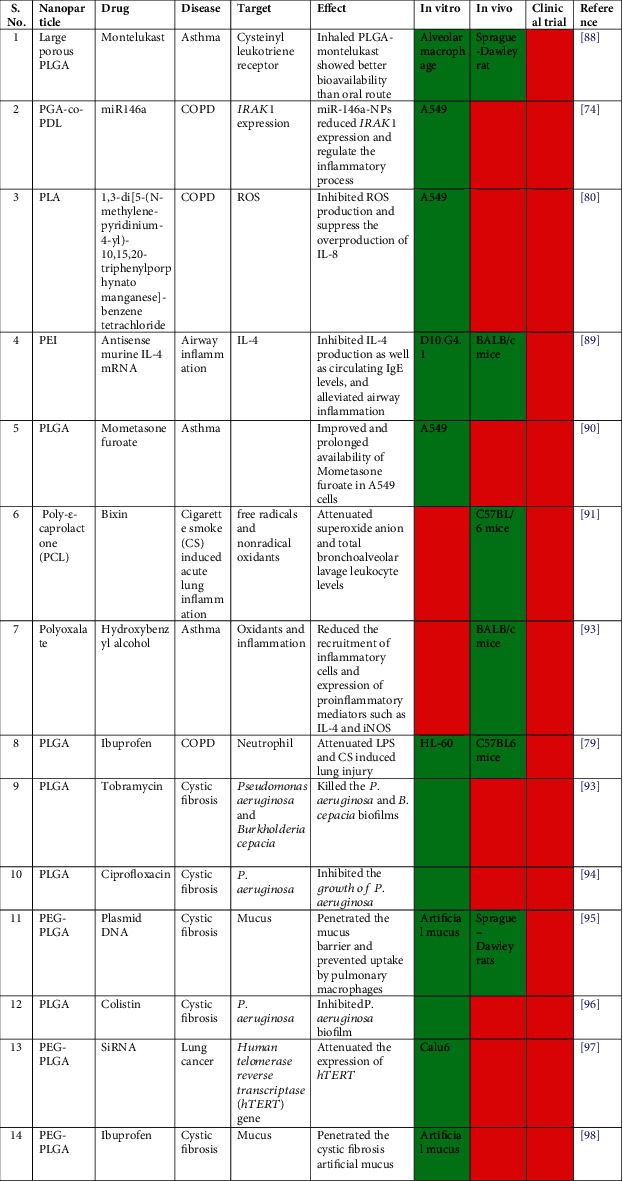
Variants of polymeric PLGA particles for drug delivery in lung diseases [73, 78, 79, 87–90, 92–97]. The green box denotes “yes” and the red box denotes “no”.

Nanomedicines conjugated with PLGA are target-specific, economical, and easy to formulate in various forms for the treatment of respiratory ailments. Despite this, very limited work on the use of PLGA-conjugated drug to combat COPD has been reported under preclinical settings (Figure 4). Interestingly, none of the PLGA NP-conjugated drug molecules showing promising results for COPD under *in vitro* and *in vivo* settings is used in clinical practice.

## 5. PLGA NPs: Prospects for COPD

Variety of synthetic (PLA, PLGA, polyacrylates, and polyanhydrides) and natural polymers (albumin, chitosan, gelatin, alginate, and collagen) are used for pulmonary disease applications. Natural polymers are not often used due to the shorter duration of drug release. Synthetic polymers are more efficient in the sustained release of the drug. Synthetic polymers are therefore routinely applied in the manufacture of NPs for drug delivery systems in lung infections [80].

Therapeutic agents are either adsorbed or encapsulated inside the core of PLGA-NPs and thereby get protected from degradation. The encapsulation led the active ingredients to cross cellular barriers and mediate the efficient release. Chemotherapeutics and molecular agents can be codelivered using PLGA-NPs. These nanoparticles exhibit a negative surface charge and get internalized by cells by clathrin-coated endocytosis or fluid-phase pinocytosis. The negatively charged surface can be altered by cationic polymers like chitosan for improved adsorption of target molecules [81].

Chronic airway inflammation and mucosa hypersecretion are a major challenge in target delivery and therapeutic bioefficacy of nano-based drug delivery systems because the nanoparticle has to bypass natural defense mechanisms and barriers. After crossing these defense mechanisms, the drugs can be targeted to diseased cells. Chronic lung obstruction in COPD is a big concern when we utilize particulate drug delivery systems. Over the last few decades, there has been growing interest in the development of targeted NPs for COPD, but reasonably less experimentation on novel drug delivery systems for chronic inflammatory and obstructive airway conditions has been carried out. Few formulations and methods for synthesizing drug-loaded nanoparticles for specific cell types in airways are currently under preclinical investigations [82].

**Figure 5:**
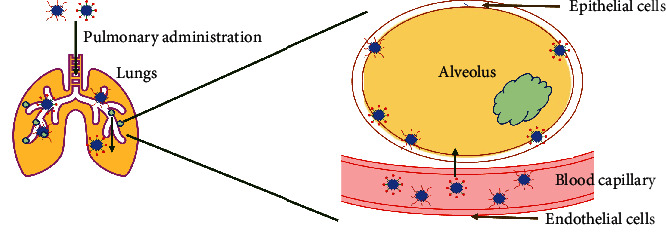
Delivery of PLGA NP-drug conjugates in COPD. Upon pulmonary administration, PLGA NP-drug conjugates reach the lungs and cross the blood capillary to move towards the alveoli.

PLGA nanoparticles have been used as particulate carriers for pulmonary inflammation and associated infections (Figure 5). Drug-loaded PLGA NPs (PLGA-PEG^PS-341^) have been initially used for slow and sustainable delivery for CF lung disease in mouse models [83, 84]. Vij (2012) also reported the potential of polymeric vesicles synthesized by a mixture of poly (ethylene glycol) and PLGA (PLGAPEG) for targeted delivery of COPD drugs like anti-inflammatory bronchodilator theophylline and the corticosteroid prednisolone [82]. For *M. tuberculosis*-associated lung infections, modified PLGA NPs encapsulated with tuftsin have shown remarkable intracellular and internalization activity in *in vitro* models. However, researchers reported having lesser diffusion in bacterial biofilms due to the hydrophobic nature of the polymer. When hydrophilic PEG polymer has been incorporated to modify PLGA NPs, there has been improved mobility of polymeric nanoparticles in human lung mucus and sputum. The controlling factors for better NPs mobility could be the density and molecular weight of PEG on the surface of particles (PLGA NPs of size 200 nm coated with PEG (Mol. Wt. 2-5 kDa) can easily penetrate through cystic fibrosis sputum; on the contrary, PLGA NPs with 10 kDa PEG exhibited mucoadhesion) [80, 85, 86].

## 6. Conclusion and Perspectives

Oxidative stress and airways inflammation contribute to severe lung pathophysiology in COPD. Also, the damage caused to the airways is poorly reversibly in COPD and the treatment options available could only minimize disease development and risk of exacerbations. Symptoms get even intensified with bacterial, fungal, or viral lung infections. Several researchers investigated the underlying molecular and cellular mechanism in COPD to devise new targets but the onset and progression of COPD only in a set of chronic smoker's population still remain a mystery. More insights and a better understanding of risk factors contributing in the pathogenesis of COPD will not only improve screening procedures but also help to evaluate improved prevention strategies. COPD is a less recognized condition and needs a bigger picture for awareness among the common to have the early diagnosis in the patients. Currently available management of COPD including reduction in exposure time to cigarette smoke, use of bronchodilators, and pulmonary restoration can temporarily relieve symptoms like improve breathlessness and exercise abilities but cannot stop the disease progression. Additionally, currently available drugs curing COPD have limited absorption and shorter half-life. Effective drug delivery systems are therefore immediately needed to treat COPD.

Biodegradable polymer drug conjugates have potential for the therapeutics of respiratory illness including COPD as an inhaled drug delivery. Polymer-conjugated drugs have better pharmacokinetic profile results in the sustained release of drug as compared with administration of drug alone. Nanotechnology-based approaches in designing drug delivery systems are found to be more effective against oxidative stress and inflammation, but the choice of selecting materials and making NPs to bring the desired bioefficacy has to be investigated in more detail. Nonimmunogenicity, preparation routes, biodegradability, absorption and release time along with clearing time and mechanisms are significantly important while selecting nanomaterials. Polymeric NPs including PLGA loaded with drug are the best available options for the COPD treatment when investigated on above parameters.

Despite several advantages, nanomedicines conjugated with PLGA have major bottlenecks like drug toxicity and narrow therapeutic efficacy. Hence, there is an urgent need to search for novel drugs, better PLGA-drug conjugates to achieve improved efficacy with minimal toxicity for effective treatment of COPD. Further research is required to formulate PLGA NP loaded with drug to monitor its drug release kinetics in the respiratory tract. This will improve the translation of PLGA-drug conjugates into clinics for effective treatment of COPD. Elucidation of the effect of PLGA NP loaded with drug on the elicitation of immune cells at the molecular level is another priority area of research.

## Data Availability

All the data in this manuscript is available with the corresponding author upon formal request.
